# Green synthesis of lead oxide nanoparticles for photo-electrocatalytic and antimicrobial applications

**DOI:** 10.3389/fchem.2023.1175114

**Published:** 2023-07-18

**Authors:** Zia Ul Haq Khan, Noor Shad Gul, Faisal Mehmood, Sana Sabahat, Nawshad Muhammad, Abdur Rahim, Jibran Iqbal, Syed Khasim, Mohamed Abdel Salam, Taj Malook Khan, Jianbo Wu

**Affiliations:** ^1^ Department of Chemistry, COMSATS University Islamabad, Islamabad, Pakistan; ^2^ Drug Discovery Research Center, Southwest Medical University, Luzhou, China; ^3^ Department of Pharmacology, Laboratory of Cardiovascular Pharmacology, The School of Pharmacy, Southwest Medical University, Luzhou, China; ^4^ Department of Environmental Sciences, COMSATS University Islamabad, Islamabad, Pakistan; ^5^ Department of Dental Materials, Institute of Basic Medical Sciences, Khyber Medical University, Peshawar, Pakistan; ^6^ College of Natural and Health Sciences, Zayed University, Abu Dhabi, United Arab Emirates; ^7^ Department of Physics, Faculty of Science, University of Tabuk, Tabuk, Saudi Arabia; ^8^ Department of Chemistry, Faculty of Science, King Abdulaziz University, Jeddah, Saudi Arabia

**Keywords:** green synthesis, lead oxide, photocatalytic activity, electrolytic properties, cyclic voltammetry, antimicrobial properties

## Abstract

Synthesis of nanoparticles (NPs) for many different uses requires the development of environmentally friendly synthesis protocols. In this article, we present a simple and environmentally friendly method to synthesize lead oxide (PbO) NPs from the plant material of the *Mangifera indica*. Analytical techniques such as spectroscopy, X-ray diffraction, and microscopy were used to characterize the synthesized PbO NPs, and their photo-electrocatalytic and antifungal properties were also evaluated. H_2_O_2_ was used to investigate the efficacy of removing methylene blue dye. At a range of pH values, H_2_O_2_ was used to study the role of hydroxyl radicals in the breakdown of methylene blue dye. Methylene blue dyes are more easily eliminated due to increased generation of the *OH radical during removal. Dye degradation was also significantly affected by the aqueous medium’s pH. Additionally, the electrocatalytic properties of the PbO NPs adapted electrode were studied in CH_3_COONa aqueous solution using cyclic voltammetry. Excellent electrocatalytic properties of the PbO NPs are shown by the unity of the anodic and cathodic peaks of the modified electrode in comparison to the stranded electrode. *Aspergillus flavus*, *Aspergillus niger*, and *Candida glabrata* were some fungi tested with the PbO NPs. *Against A. flavus* (40%) and *A. niger* (50%), and *C. glabrata* (75%), the PbO NPs display an excellent inhibition zone. Finally, PbO NPs were used in antioxidant studies with the powerful antioxidant 2, 2 diphenyl-1-picrylhydrazyl (DPPH). This study presents a simple and environmentally friendly method for synthesizing PbO NPs with multiple uses, including photo-electrocatalytic and antimicrobial activity.

## Introduction

The emerging era of nanotechnology has swiftly impacted various medical, environmental, solar energy, and pharmaceutical industries, where metal-based nanoparticles (NPs) are being used as an efficient material compared to their bulk counterparts (Miri et al., 2018; Sutjaritvorakul and Chutipaijit, 2020). Due to their extremely small size, NPs have a large surface area to volume ratio and multiple dimensions, which shows more flexible properties than bulk materials (Elango and Selvaraj, 2015). For instance, air and water are purified using different methods to eliminate the pathogenic microbes and toxic organic compounds (Oszlánczi et al., 2011; Murthy and Vijayaragavan, 2014; Diallo et al., 2015; Miri et al., 2018). Anatase TiO_2_ has been widely demonstrated as anti-bacterial material which eliminates the bacteria by oxidation (Gerard et al., 2006; Narayanan, 2012; Vallinayagam et al., 2021). Similarly, PbO is extensively used in ceramics, pigments, glass, gas sensors, and battery manufacturing industries. PbO is prepared in various methods, such as chemical, physical and biological synthesis in different shapes and dimensions (Omidtorshiz et al., 2023). Among the different methods, green synthesis of PbO NPs has gained huge interest due to its simple and sustainable characteristics, which use non-toxic reaction media and solvents without affecting the environment (Szymanski and Dobrucka et al., 2023). Methylene Blue is a common dye found in wastewater that is degraded or deactivated with noble metal NPs to eliminate the disease-causing bacteria and breakdown MB via Reactive Oxygen Species (Çetİnkaya and Kütük, 2023). In photocatalytic studies, malachite green dye (Murthy and Vijayaragavan, 2014; Palani et al., 2022). Despite this, MNP’s are likely to accumulate and have low strength. Zeolite, Fe_2_O_3_, TiO_2_, and graphene oxide have all been employed as support for nanoparticles to avoid the aggregation and solve the separation, stability, and recovery issues associated with MNPs (Nasrollahzadeh et al., 2014; Nasrollahzadeh et al., 2015a; Nasrollahzadeh et al., 2015b; Tahir et al., 2016). The PbONPs were used to obtain highly scatter able, deeply uncovered, and extremely large surfaces of small-size nanoparticles (Noukelag et al., 2021). PbO NPs are widely utilized as efficient supports for organic reactions because of their high thermal and chemical strength, optical properties, minimal expense, and low toxicity, in addition to their high photocatalytic movement and reproducibility (Chen and Mao, 2007). As part of nanotechnology, biosynthesized nanoparticles play a significant role. Nobel metal nanoparticles have been synthesized using fungi (Balaji et al., 2009; Fayaz et al., 2009; Huang et al., 2013), bacteria (Ahmad et al., 2003), and plants (Khan et al., 2016a). The dispersion, size and shape significantly influence the biological, physical and chemical properties of the NPs. The biosynthesis of metal oxide nanoparticles improves their physical, biological, and chemical characteristics, thereby minimizing hazardous by-products.

Herein, we have developed a green plant extract-based synthesis of PbO for multiple materials for photo-electrocatalytic and antifungal applications. *Magnifier indicia* plant extract was used as a reducing agent for synthesizing PbONPs.

## Materials and methods

### Collection of plant

The plant sample of Magnifera indica was collected from Kot Addu, Punjab, and washed thoroughly with clean water. Further, the material was dried at 25°C–30°C, and subsequently, the remaining plant matter was reduced to a powder. To prepare plant extract, 20 g of biomass was dipped in 200 mL water with constant stirring. After a final filtration, the filtrate from the plant extract was utilized to synthesize PbO NPs (Omidtorshiz et al., 2023).

### Synthesis of PbO NPs

**SCHEME 1:**
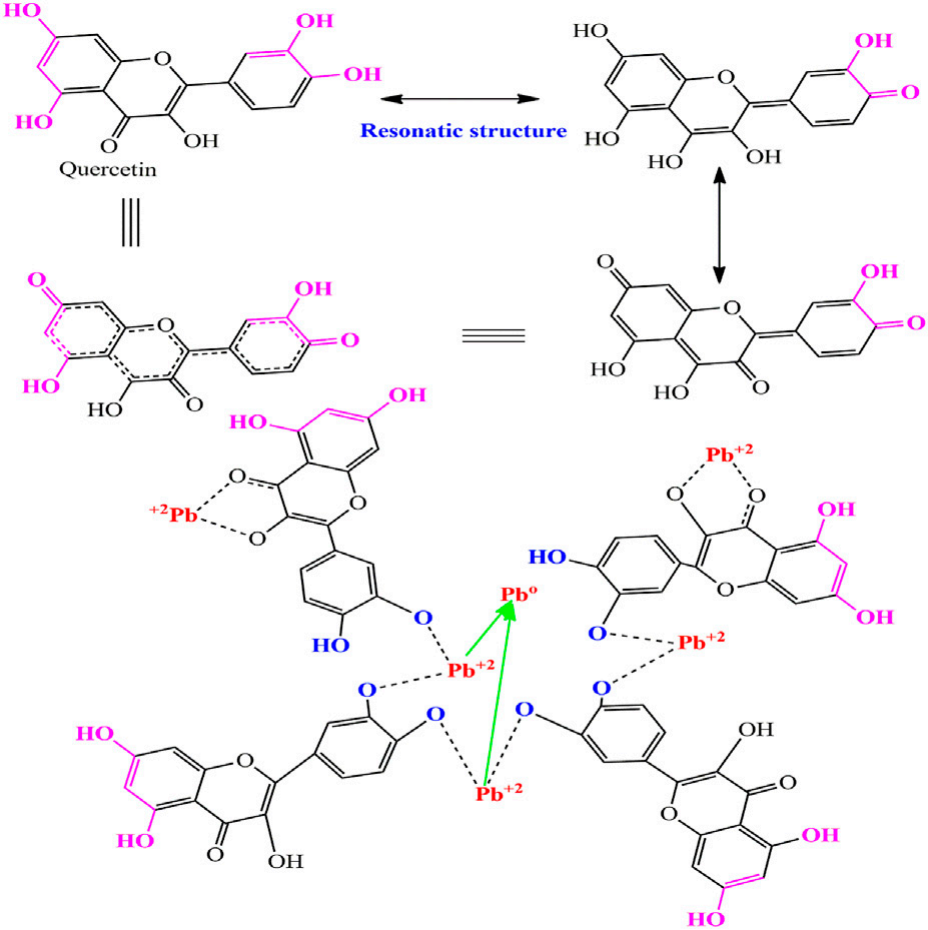
Schematic proposed Reduction of Pb^+2^ to Pb^0^ through bioactive organic compounds.

For the synthesis of PbO NPs *Magnifier indicia* biomass was mixed with 50 mL of 6 × 10^−3^ M solution of PbCl_2_ stirring (Tahir et al., 2016; Omidtorshiz et al., 2023). During the synthetic process, the greenish color changed to the blackish of the mixed solution. In addition, plasmonic peak and synthetic procedures of PbO NPs were investigated using a UV/Visible spectrophotometer (Khan et al., 2016a). After confirming the PbO NPs with ultraviolet light, they were centrifuged for 15 min at 5,000 revolutions per minute. Moreover, the PbO NPs were collected from the wall of the tubes after centrifugation. Then the phytochemicals of *Mangnifera indica* were used for the redox of ${Pb}^{2+}$ to ${Pb}^{0}.$ As a result, active constituents (*Quercetin*) of *Magnifier indicia* stabilized metallic ions to zero-valent metal (Palafox-Carlos et al., 2012; Zahoor et al., 2020). Due to phenolic compounds, *Magnifier indicia* oxidized quickly through autoxidation of Pb^2+.^The redox of ${Pb}^{2+}$ to ${Pb}^{0}$ through phytochemicals is presented in Scheme 1.

### Photocatalytic activity

The photocatalytic properties of PbONPs under UV/visible were studied for the MB degradation (Tahir et al., 2015a). Furthermore, the PbO NPs of 10 g were added with 15 mL of MB solution to investigate the photocatalytic activities (Tahir et al., 2015c). Monochromatic emanation at 245 nm Hg lamp of lower pressure was used as a light source. A Methylene Blue solution was used to determine the photocatalytic activity of lead nanoparticles. Different concentrations of PbONPs over time intervals were used to investigate MB’s photocatalytic degradation. Lead nanoparticles’ photocatalytic activity was evaluated using the elimination of MB dye as a model (Khan et al., 2021). MB dye solution and PbO NPs (8 mg) were mixed in 15 mL (Khan et al., 2021). After preparation, the solution was exposed to UV/Visible light to study the photocatalytic degradation of MB at 10 min. Interval. The following Eq. 1 was used to calculate the removal of MB.

$$Degradation\;\left(\%\right)=\left(\frac{A_{c}-A_{t}}{A_{c}}\right)\times 100$$ (1)

*Where A*_*c*_ represents the absorbance of MB (without PbO NPs), *A*_*t*_ represents the absorbance of the test solution (MB & PbO NPs). The degradation of MB was studied in parallel with a blank solution under UV light.

### Cyclic voltammetry analysis

To evaluate cyclic voltametric data, the CS-300 and 150 workstations were employed. In order to evaluate CV, a standard three-electrode protocol was used. Glassy carbon (GC) and GC@PbO NPs were used as the modified working electrode. Platinum and SCE was used as counter and retraces electrode, respectively.

### Electrochemical study of modified PbO NPs electrode

Glassy Carbon was polished several times with alpha Al_2_O_3_ of different sized (0.5–0.05 mm and washed in CHO_3_H) (Salunke et al., 2018). To prepare the modified electrode, the polished GC electrode was immersed in CH_3_OH suspension containing 3.5 g of PbONPs and activated carbon at 25^C^ (Khan et al., 2016f). As a further matter, the PbO/@GC modified electrode was washed through clean water to remove less bounded PbO NPs. Finally, the redox reaction was calculated through cyclic voltammetric analysis of GC/PbO NPs modified electrode.

### Electrode preparation

For the preparation PbONPs electrode, 0.6 g biochar (activated carbon) was taken in a 100 mL beaker. Furthermore, 1 g of PbO NPs was measured in a container containing C_2_H_4_O. [27, 28]. Polytetrafluoroethylene (PTFE) 0.2 g was used as a binding solvent. After washing, the materials were packed in a small plastic sheet and dried in an oven under different temperatures for 2–3 h [27]. Moreover, a small plastic bag was refrigerated for 24 h at 120°C, and the electrode potential of the materials ware studied.

### Antibacterial activity

The antimicrobial properties of PbONPs were investigated through the Agar Well Diffusion process (Ahmad et al., 2016). For streaking, the inocula of *E. coli, B. subtilis, S. aureus, and S. typhi* using Muller Hinton Agar was spread in petri dishes to confirm even lawn on strained growth. Through sterile cork, 8 mm wells were bored in the PD. Forbye, the green synthesized PbONPs were kept in plates at 25°C, and the diameter of inhibition was calculated after 24 h. Interestingly, the PbO NPs showed excellent activity, as documented in the literature (Azarang et al., 2018; Tailor and Lawal, 2021). Each experiment was conducted in triplicate, and the response of the PbONPs was reported as means ±SD (Khan et al., 2016c). Clarithromycin was used as a standard for the sake of understanding, and the control comprised 100μL and 200 μL of DMSO (Khan et al., 2016c).

### Production ROS through PbO NPs

**SCHEME 2:**
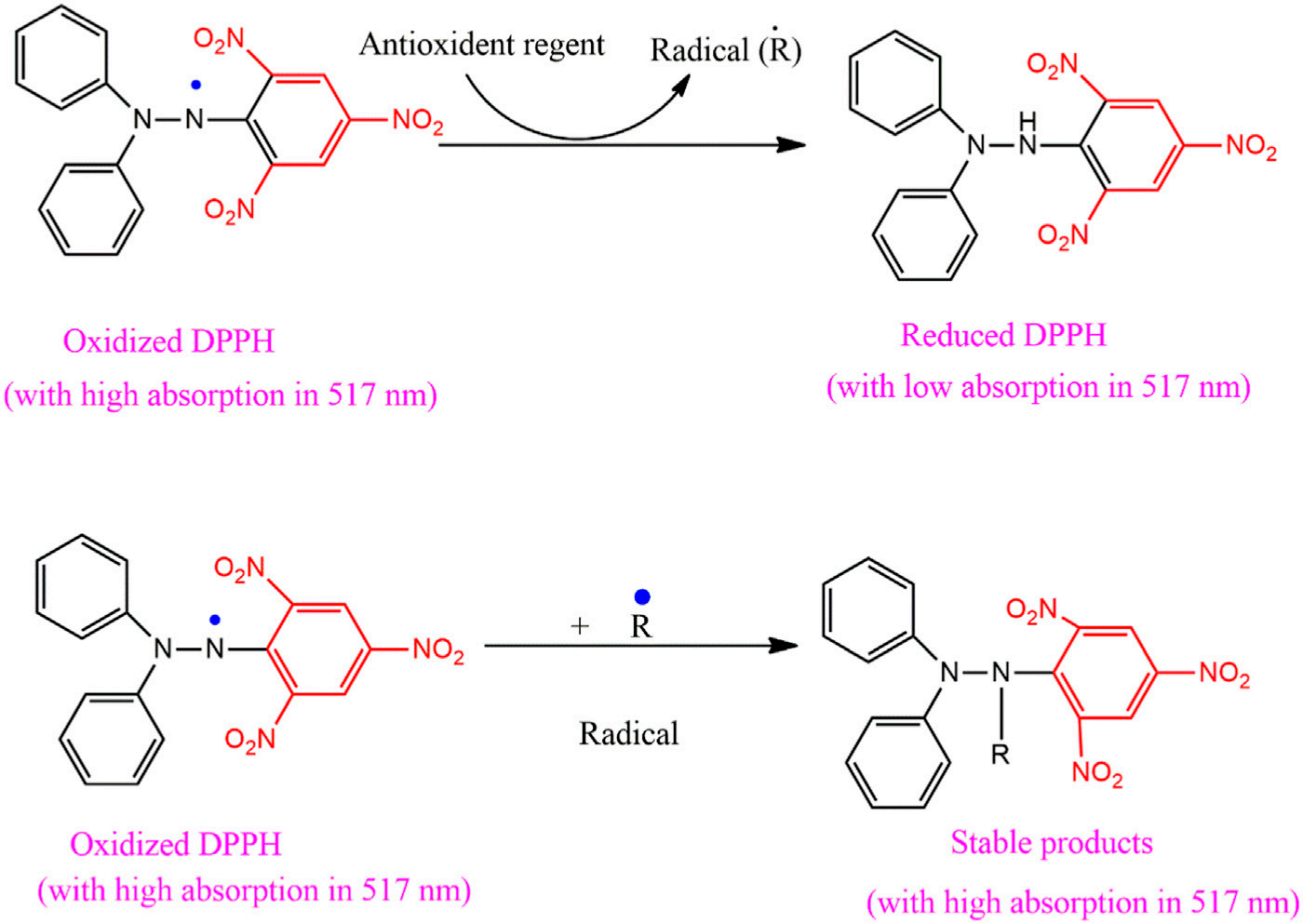
Mechanism of DDPH with absorption in 517 nm.

To recognize ROS (•OH) in the cell’s body, 2, 7-dichlorodihydrofluorescein diacetate (DCFH-DA) was used as an indicator. Antimicrobial activities of PbONPs were studied against bacterial strains. Further, the suspension of the pellets in 1 mL solution was conserved with 1 mM DCFH-DA reagent for 30 to 40 mines. Finally, the buffer solution was used to eliminate excess organic pollutants/dyes from the cell surface (Li and Shah, 2003). Schematic representation of Production of ROS shown in Scheme 2, S1 (supporting data).

### Antifungal assay

To determine the antifungal activities of the green synthesized PbO NPs, Sabouraud dextrose agar was used. For the purpose of assessing antifungal activity, *Aspergilus flavus* and *Aspergius niger* was used for the fungal strain study (Khan et al., 2016c). After autoclaving, 100 μL of NPs, the suspension was transferred into the tubes and reserved in a driven location. To determine inhibition evolution, 5 mm of the fungal colony was retained (Tahir et al., 2015b). Eq. 2 was applied to estimate mycelial growth (%) from the fungal growth area (cm^2^) (Khan et al., 2016c). Moreover, an open beaker of water was placed in the incubator to control the humidity for 40%–50%. Consequently, linear growth was calculated (mm) with reference to the negative control (Khan et al., 2016b).

$$I\;\left(\%\right)=\left(\frac{G_{c}-G_{t}}{G_{c}}\right)\times 100$$ (2)

In Eq. 2, the growing of mycelia (control) is shown by *Gc,* and the mycelia development with the action of PbONPs was determined by *Gt*.

### Anti-oxidant activities

The antioxidant properties of PbONPs were calculated through DPPH. Then, test models were placed in the dark at 37°C for 1 hour and used ascorbic acid as a reference. Different concentrations of solutions 100, 250, 500 and 1000 µL were made after mixing almost 900 µL of DPPH solution with 100 µL of the test sample (Khan et al., 2016c). Negative control was taken as a combination of 100 µL 3% CH_3_OH sample and 900 µL of DPPH, and methanol 3% was used as a blank solution (Khan F. U. et al., 2016). Antioxidant activity was determined through Eq. 3.

$$Inhibition\;\left(\%\right)=\left(\frac{A_{c}-A_{t}}{A_{c}}\right)\times 100$$ (3)

## Results and discussion

### UV-visible spectroscopy

**FIGURE 1:**
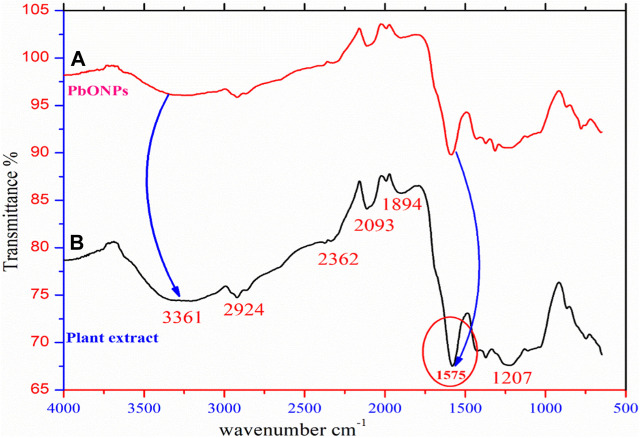
**(A)** FTIR spectra of green synthesized PbONPs. **(B)** FTIR spectra of plant extract.

UV/VIS spectra, as shown in Supplementary Figure S1, provided conclusive evidence for the biosynthesis of PbO NPs. In addition, the outer shell en absorbed external energy and subsequently jumped to a higher energy level (HOMO to LUMO). As Pb^+2^ was reduced into Pb nanoparticles, a color change occurred in the exposed plant extract. The blue shift was due to surface plasmon resonance (SPR), and the SPR absorption band at 275 nm is due to free electrons in metal NPs. In addition, bright visible spectroscopy can quantify strain; the formation can reveal strained particles via a corresponding change in spectra by rakish twisting, and it can be used to differentiate tautomeric structures. UV-Visible spectrometer readings were taken regularly from aliquots of the photosynthesized PbO NPs to ensure quality. Moreover, the peak of the biogenic PbO NPs’ SP was identified using UV-Visible spectroscopy. Due to the SPR peak depicted in Figure [1], the conducting electron oscillates at certain wavelength ranges. Materials particle size and shape for NPs synthesis and Pb reduction are all influenced by the % -OH and other active bioactive constituents. These hydroxyl compounds lowered the concentration of M+ and kept it there [14]. In addition, the SPR is affected by the size, shape, and distribution of the PbO NPs. References in the literature support the presence of PbO NPs in the reaction mixture, as evidenced by the prominent SPR peak at 352 nm (Tabassum et al., 2013; Usha et al., 2015; Tahir et al., 2016).

### FT-IR analysis

Figure 1A, B shows the 4,000–400 cm^−1^ wavenumber region of the FTIR spectra of PbO NPs with a biological target. In addition, the peak at 3,361 cm^−1^ indicates the stretching vibration of the -OH bond, confirming the alcoholic or phenolic nature of the biomass. Between 2,939 and 2,362 cm^−11^, the -C-H str-vibr-peak could be observed. In the range of 1894 cm^−1^, -C-H stretching is visible, and the peak at 2093 cm^−1^ is associated with the -C=C- stretching vibration (Khan et al., 2016a; Khan et al., 2016c; Shah et al., 2018). Moreover, the C=O stretching vibration of the keto and carboxyl groups is associated with the 1,575 cm^−1^ peak. Additionally, the -C-O-H bending and C-C bonds extended their vibrations at 1,207 cm^−1^ (Mobarra et al., 2016). Therefore the results of this study show that plant extract biomolecules like flavonoids and phenolic acids were used in the synthesis and stability of nanomaterials, as evidenced by a decrease in peak intensities.

### XRD analysis

**FIGURE 2:**
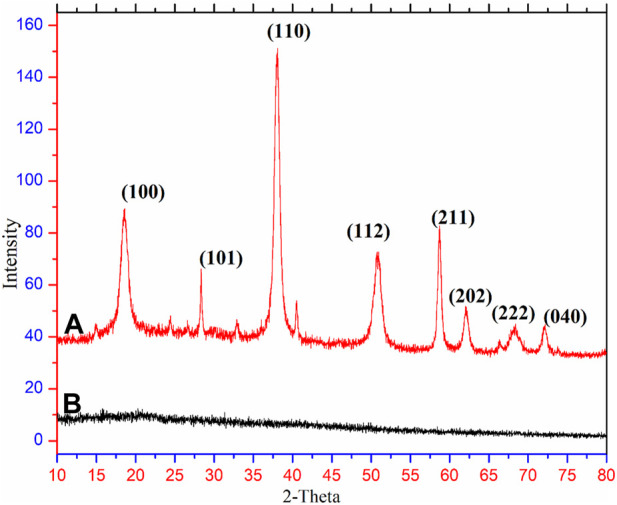
XRD of green synthesized PbONPs **(A)**, Plant extract **(B)**.

XRD analysis confirms the crystalline structure of PbO NPs, as shown in Figure 2. XRD occurs in the lattice planes at a typical angle in a sample. XRD at 10^o^-80^o^ values interpreted the confirmation of PbO NPs crystalline. On top of that, Bragg’s reflection numbers at 2theta are 100, 101, 110, 112, 211, 202, 222, and 040 in lattice planes, respectively. Additionally, the main expansion of PbO NPs along the direction of (110) is suggested by the spectrum peak intensity for (110), which is significantly higher than the other pattern in the lattice structures plane (Gibson et al., 2011). PbO NPs were very pure, as evidenced by their XRD pattern (Güngör et al., 2017). Corroborated by the HRTEM images, the average particle size calculated using the Debye-Scherrer equation (Eq. 4) was 50 nm (Holzwarth and Gibson, 2011; Mallick and Dash, 2013; Deotale and Nandedkar, 2016).

D=0.94 λ/ß⁡cos⁡θ (4)

Where *D* stands for the size of the particle, *k* stands for the shape factor, the equation’s constant (also known as the Scherer constant value) has values ranging from 0.9 to 1, *β* denoted as ∆ (*2θ*), is expressed as the full-width half maximum in radian λ source of X-ray wavelength and θ is the Bragg angle.

### SEM and EDX analysis

**FIGURE 3:**
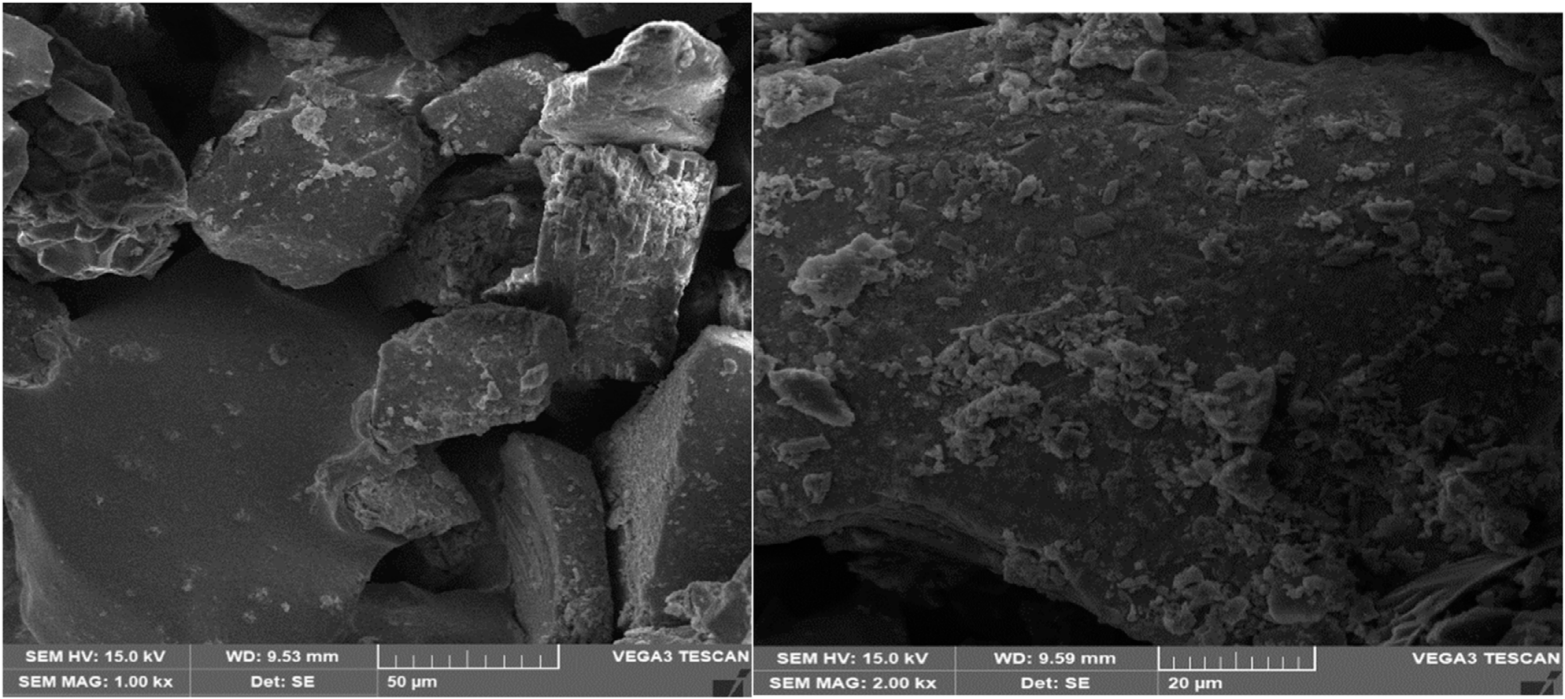
SEM Images of green synthesized PbONPs.

The surface, size and geometry of biogenic PbO NPs are shown in Figure 3. Moreover, the activity directly affected the size of the particle. Interestingly, the particles of smaller size and larger surface area showed good activities. In contrast, smaller-sized particles showed the best absorbing activity of the existing dyes in contaminated wastewater (Khan et al., 2016e).

EDX was used to determine and confirm the elemental analysis of PbONPs, as shown in Supplementary Figure S2. Furthermore, the EDX determined that the lead solution reduction with *Magnifier indicia* plant extract resulted in the synthesis of crystalline PbO NPs (Mahmoud et al., 2018). Strong Pb, Carbon, and Oxygen peaks showed the above elements’ availability at the lead’s surface (Yaqoob et al., 2021).

### Histogram

The crystalline size of the PbO NPs nanoparticles was determined through histogram using ImageJ software (Khan et al., 2022), as shown in Supplementary Figure S3. Histogram analysis verified the particle size and distribution of PbO NPs. PbO NPs were found to have an average particle size between 0.6 and 85 nm.

### Electrochemical behavior

**FIGURE 4:**
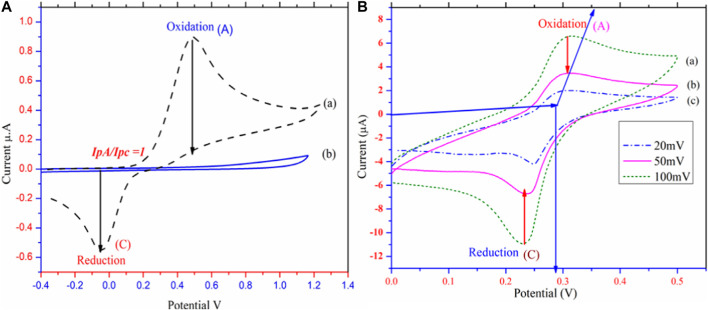
Cyclic voltammetric studies **(A)** Cyclic voltammetric investigation of Glassy Carbon (Reference) **(B)**. Cyclic voltammetric investigation of @PbONPs in the presence of sodium acetate as electrolyte in room temprature at different scaning rate vs SCE mV^−1^.

Electrochemical studies of PbO NPs were investigated viacyclic voltammetry in astrong electrolyte solution ofsodium acetate. **7a-b** (Khan et al., 2016f). Carbon Electrode (GCE) cyclic voltammetry was investigated, and the results are shown in Figure 4A (Nematollahi and Forooghi, 2002; Luo et al., 2012). Because **1a** is a constant at the electrode surface, Figure 4A displays both anodic and cathodic peaks, and the IpA/IpC is equal to unity (Stojanović et al., 2016). During the electrochemical process, 1,4-dihydroxybenzene (**1a**) was oxidized to the reactive species quinone (**2a**), a newly generated species is stable near the electrode’s surface. Green synthesized PbO NPs/@GC paste electrode cyclic voltammetry was studied in an aqueous system with 0.15 M C_2_H_4_O as the supporting electrolyte. This was because the Ip_A_/Ip_C_ ratio at the paste electrode was not unity during the redox reaction. At 0.35 V, the anodic peak A is observed. As can be seen in Figure 4B, the modified electrode was used as the working electrode, and 1-4-dihydroxy benzene was oxidized on its surface (Abdelmalek et al., 2006). Low-intensity appearance of the anodic and cathodic peaks during the redox process is evidence that reactants are being converted into products. Different scanning (20,50 and 100 V) at 25°C have been used to examine the scanning effect of the modified electrode. Scheme 2 S2 depicts the synthesis of quinone **(2a)** from 1,4-dihydroxy benzene **(1a).**

### Removal of methylene blue dye through H_2_O_2_

**FIGURE 5:**
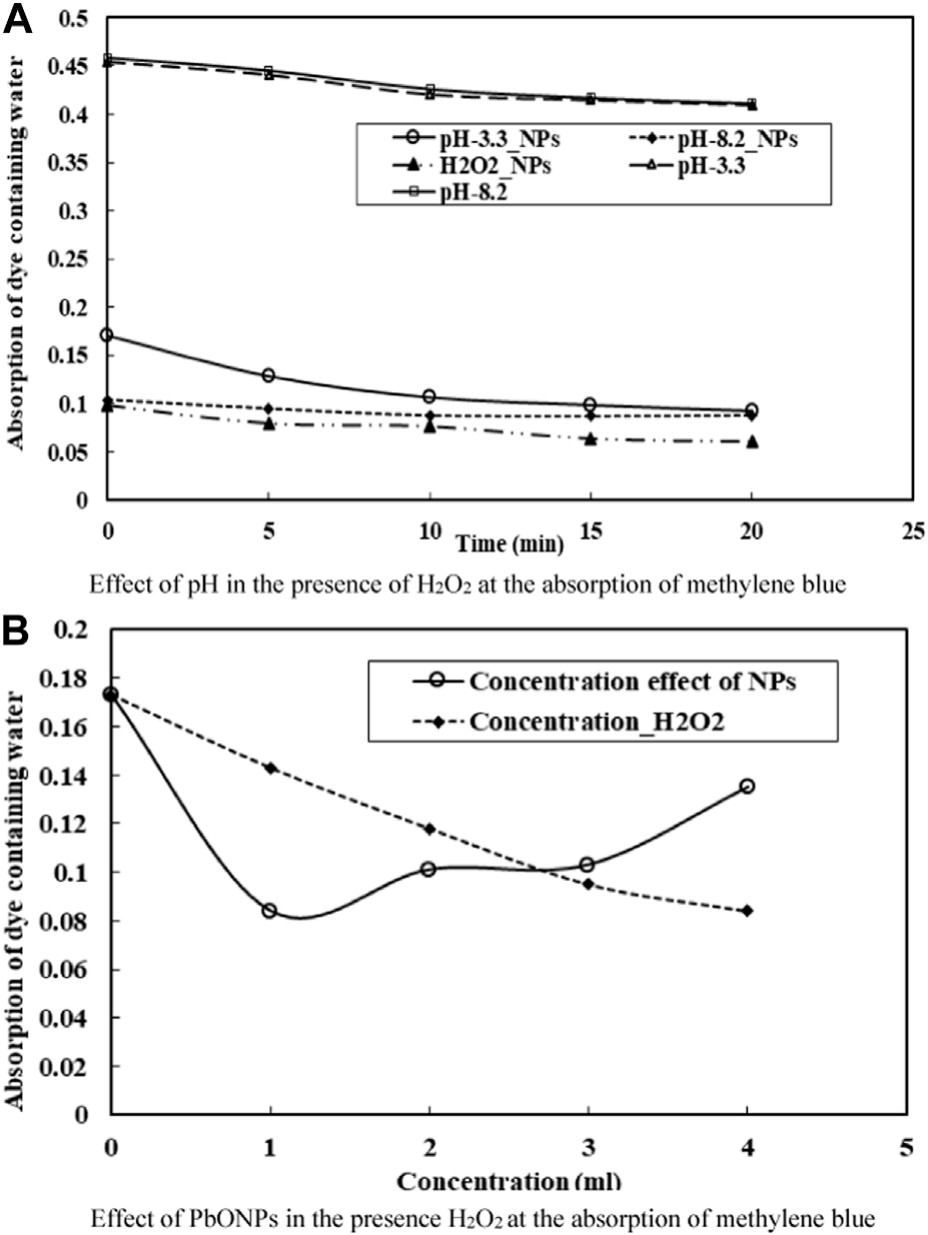
**(A)** Effect of pH in the presence of H_2_O_2_ at the absorption of methylene blue. **(B)** Effect of PbONPs in the presence H_2_O_2_ at the absorption of methylene blue.

According to the research, H_2_O_2_ is very important in decomposing organic pollutants. Removal of MB dye was investigated as a function of H_2_O_2_ concentration. Figures 5A,B depicts the role of H_2_O_2_ in MB deterioration. The reaction begins with the formation of hydroxyl radical species. In both acidic and basic media (i.e., 3.2–8.2 pH), H_2_O_2_ was used to study the role of ^●^OH radicals in eliminating MB dye while the dye concentration remained constant. Findings suggested higher H_2_O_2_ concentrations were associated with more efficient MB dye removal. It has been discovered that the removal of MB is significantly aided by an increase in the generation of the Hydroxyl radical in response to an increase in H_2_O_2_ (Shah et al., 2018). H_2_O_2_ concentrations directly affect the efficacy of organic pollutant removal (Shah et al., 2018; Iqbal et al., 2020). Moreover, the production of ^●^OH radical in Fenton-reaction acts as a strong oxidizing agent and an electron scavenger (Shah et al., 2020). Finally, the following reactions have been proposed to account for MB’s decomposition: (Eqs 5–11).

$$PbO\;\left(h_{VB}\right)+H202\to PbO{+}^{•}H\;{+}^{•}OH$$ (5)

$$PbO{\left(h_{vb}^{+}\right)}^{•}{+}^{•}OH\;\to PbO{+}^{•}OH$$ (6)

$$PbO\left(e_{CB}^{-}\right)+O_{2}\to PbO+O_{2}^{.-}$$ (7)

$$O_{2}^{.-}+H^{+}\to {HO^{•}}_{2}$$ (8)

$$Dye+{HO^{•}}^{.}\;\to Degradation\;products$$ (9)

$$Dye+h_{VB+\to Oxidation\;Products}$$ (10)

$$Dye+e^{-CB}\;\to Reduction\;Products$$ (11)

In this finding, we gazed at how H_2_O_2_ and the catalyst PbO NPs affected the removal of MB. According to the investigation, PbO NPs’ catalytic efficiency increased dramatically upon H_2_O_2_ addition. It was found that the reactive species have a high redox potential (2.8V) and react vigorously with the target contaminants (Shah et al., 2020). Supplementary Figure S4 depicts a potential pathway for the photocatalytic degradation of Methylene Blue.

### Zeta potentials

Through Zeta potentials, the PbO NPs appeared to be smaller and more spherical, as shown in Supplementary Figure S5. In addition, the Zeta Potential value of 34.1 mV for the newly biosynthesized lead oxide nanoparticles demonstrates their increased surface area. In addition to increased stability, reactivity, and dispersion, a greater zeta potential value confirms that bioactive species surround NPs, boosting the force of repulsion between PbO NPs and preventing the aggregation process (Khan et al., 2018).

### Antibacterial activates of PbO NPs

The results of screening several extracts against various pathogenic microorganisms are shown in Table 1. *Escherichia coli* was inhibited by the PbONPs up to a maximum of 36%. *S. typhi* was the least inhibited, and *E. coli* was the most inhibited. Maximum *E. coli* inhibition by the PbONPs was 36% (Feng et al., 2015; Ullah et al., 2019). According to a WHO survey, approximately 43% of all deaths worldwide are attributable to infectious diseases (Boutayeb et al., 2013). There must be a constant push for the development of new efficient, and harmless antimicrobial drugs. To kill bacteria, NPs must come into physical contact with the microbes. Next, NPs interact with DNA, lysosomes, ribosomes, and enzymes, the fundamental components of bacterial cell walls. The release of reactive oxygen species (ROS) is a crucial part of NPs’ antibacterial mechanism. It is important to note that different types of NPs have different effects on oxygen molecules, leading to a wide range of ROS. Free radicals and ROS refer to molecules and reactive intermediates with positive redox potential. In patients with compromised immune systems, such as cancer or other tumors, drug-resistant microorganisms complicate the treatment of infectious diseases (Rogers, 2022). In patients with compromised immune systems, such as cancer or other tumors, drug-resistant microorganisms complicate the treatment of infectious diseases (Rogers, 2022). Many effective antibiotics have been isolated from naturally occurring medicinal plants, which has aided in the search for novel antimicrobial compounds (Martini et al., 2004).). Among tannins’ many uses, antibacterial and astringent properties are particularly well-known. This confirms what was discovered in the prior study (Sunilson et al., 2009). Supplementary Figure S6 depicts the hypothesized relationship between ROS production by PbONPs and subsequent cell damage.

**TABLE 1:** Antibacterial activity.

Bacteria	Z.I standard (Clarithromycin)	% Inhibition
		PbONPs
*E. coli*	29	36
*S. aureus*	33	16
*S. typhi*	22	7

Z.I = zone of inhibition in mm, Inh = inhibition in Percent (%), The plates were inoculated at a concentration (mg/mL) of DMSO. b = Clarithromycin, MIC= minimum inhibition concentration.

## Antifungal activates of PbO NPs

PbONPs’ antifungal properties are shown in Table 2. According to the results, PbO NPs inhibited the growth of *Aspergillus flavus* (40%), *Candida glabrata* (75%), and *Aspergillus niger* (50%) (Trivedi et al., 2022). PbO NPs suppressed *Aspergillus flavus* growth by 40%). The PbONPs demonstrated effective suppression of the human allergen *A. Flavus*, which is the cause of dermatophytosis and dermatophytosis. The PbONPs were active (30%) against *A. niger*, resulting in a fungal ear infection and, in extreme circumstances, harm to the tympanic membrane and ear canal (Hilton, 2018).

**TABLE 2:** Antifungal activity.

Fungi	Standard drug^a^ (MIC μg/mL)	PbONPs	
		L.G	Inhibition
*Aspergillus flavus*	105	30	40
Aspergillus niger	30	50	50
*Candida glabrata*	75	40	75

L.G = linear growth in mm, Inh = inhibition in Percent (%), The plates were inoculated at a concentration (mg/mL) of DMSO. a = Terbinofin (standard drug), MIC = minimum inhibition concentration.

### Antioxidant properties of PbO NPs

The powerful antioxidant activity of the PbONPs against DPPH is shown in Supplementary Table S1; (Patel Rajesh and Patel, 2011). Mild DPPH-scavenging effects were reported for PbONPs, which demonstrated antioxidant activity (Patel Rajesh and Patel, 2011). Photosynthesized NPs were studied for their ability to scavenge free radicals. Heart disease, diabetes, atherosclerosis, arthritis, severe infections, nervous system syndromes, cancer, immune system destruction, pain, and discomfort can all be traced back to reactive oxygen species, also known as free radicals. Antioxidant drugs are developed and validated using a variety of technologies to demonstrate their biological reactions against toxins, such as peroxides decomposition, reduction capacity and hydrogen generalization, chelating of transition metal ions, inhibition of chain initiation reaction, and prevention of scavenging radicals (Ahmad et al., 2016; Khan et al., 2022). Antioxidant therapies are measured for their potential to impact biological activity using DPPH.

## Conclusion

In this study we report *Mangnifera indica* plant material as a stabilizing material to synthesize PbONPs. Superior stability, monodispersity, and controlled size distribution were all features of biosynthesized PbONPs. PbONPs induced apoptosis (programmed cell death) in bacteria by interacting with peptidoglycan, which caused structural changes in the peptidoglycan. PbO NPs were tested for their antimicrobial and antifungal properties. Despite having a lower potential and sensitivity, PbO NPs showed exceptional electrochemical and electrocatalytic capabilities. Reduction of alcoholic and phenolic compounds, as well as other reactions, can be accomplished with great success using the PbO NPs/GC in its modified form. Synthesis of PbO NPs follows an environmentally benign process that does not involve the use of any toxic chemicals. The findings indicated that biosynthesized PbO NPs have great potential for biomedical and electrocatalytic applications. Deeper research is needed to pave the way to uncover PbONPs’ hidden perspectives in the world.

## Data Availability

The raw data supporting the conclusion of this article will be made available by the authors, without undue reservation.
