# Supplementary material for: Green synthesis of lead oxide nanoparticles for photo-electrocatalytic and antimicrobial applications
Source: Front Chem. 2023 Jul 18;11:1175114. doi: 10.3389/fchem.2023.1175114 (PMC10435987; doi:10.3389/fchem.2023.1175114)
Supplement: Supplementary file 1 [file DataSheet1.docx]

**Supporting Data**





**Figure S1: UV spectra of biosynthesized PbONPs**


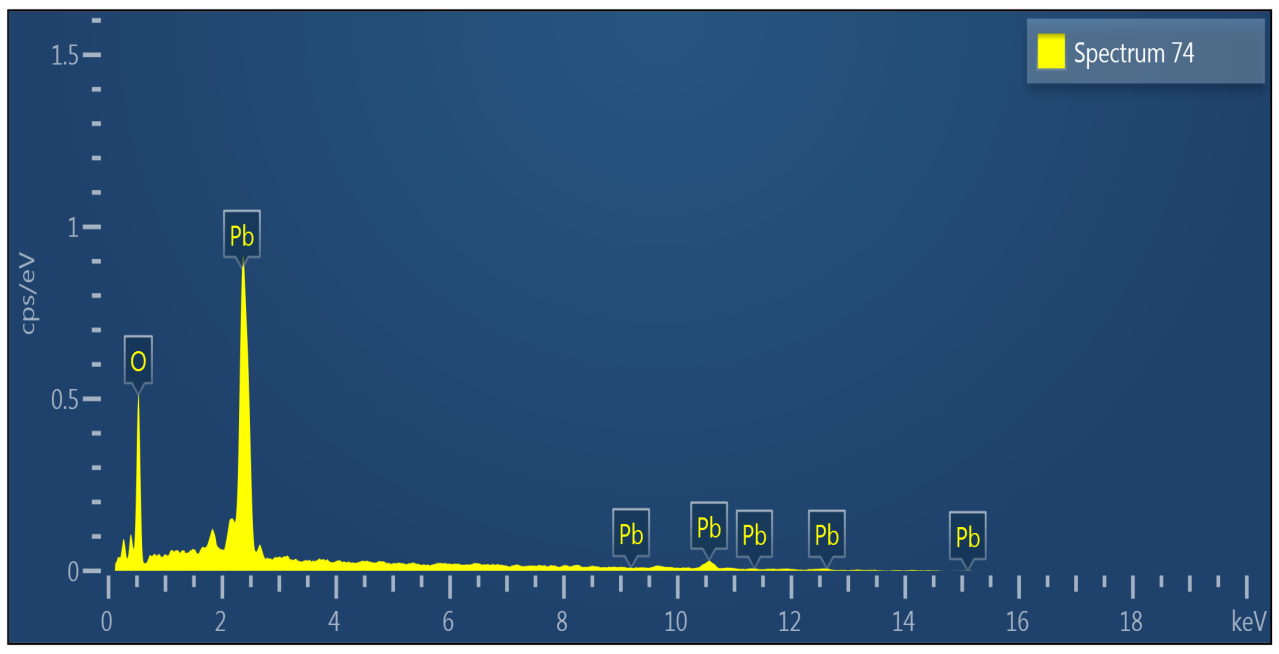


**Figure S2 :EDX Images of green synthesized PbONPs**





**Figure S3: Histogram spectrum of PbONPs**


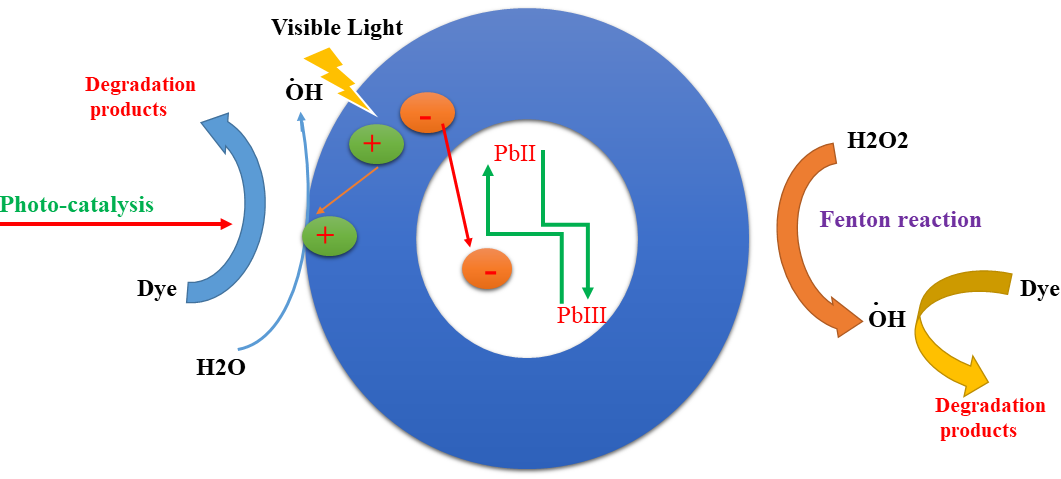


**Figure S4:** Proposed photo catalytic degradation of Methylene blue through Fenton type reaction.


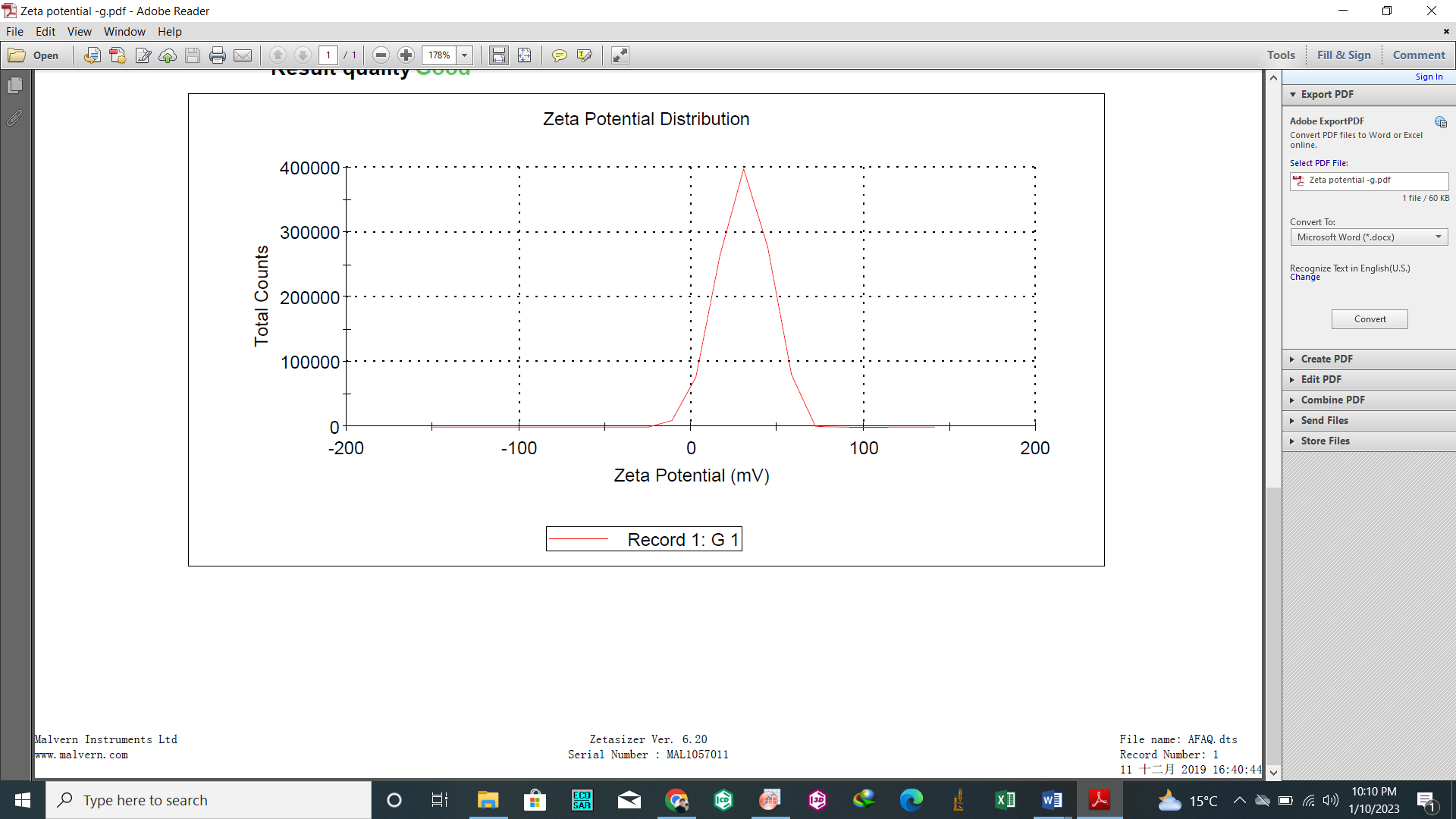


**Figure S5:** Zeta potential of the biosynthesized PbONPs


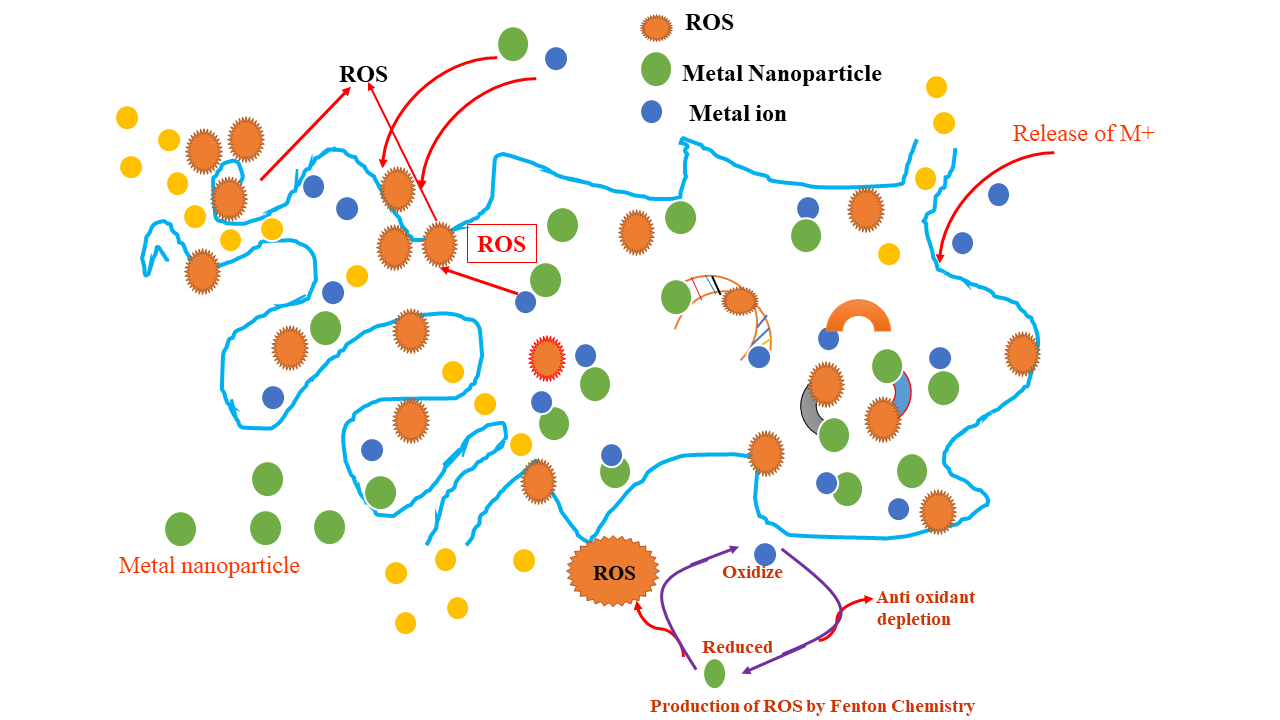


**Figure S6:** Proposed mechanisms of ROS generation from PbONPs and their cell damage response





**Scheme 2, S1**: s**chematic representation of Production of ROS**





**Scheme S2 :**Electrochemical redox reaction of hydroquinone

Table S1 : Concentration-dependent DPPH free radical scavenging effects

| Concentration (μg/ml) | Standard drugs | (a) Concentration-dependent DPPH free radical scavenging effects of E. vesicaria control and affected on Vas (Penicillin) |
| --- | --- | --- |
|  | **Ascorbic acid** | **PbONPs** |
| 25 | 40.2±0.6 | 25.1 ± 0.11 |
| 50 | 95 ±0.1 | 47.5± 0.14 |
| 75 | 100 | 85.5± 0.17 |
| 100 | --------- | 90.3 ± 0.31 |
| IC_50_, (μg/ml) | 35 ±0.04 | 70.04± 0.4 |

%DPPH free radicals scavenging effect= (DPPH Ab-sample Ab/ DPPH Ab) X 100

Values shown are mean ± SEM, no. of experiments =
